# A scoping review of women’s experiences and barriers in automated vehicle research

**DOI:** 10.1371/journal.pone.0331402

**Published:** 2025-09-10

**Authors:** Alaa H. A. Abusafia, Alessandro Soro, Ronald Schroeter

**Affiliations:** 1 Faculty of Science – Queensland University of Technology (QUT), Brisbane, Queensland, Australia; 2 Faculty of Health – Queensland University of Technology (QUT), Brisbane, Queensland, Australia; Gannon University, UNITED STATES OF AMERICA

## Abstract

Automated vehicles (AVs) have the potential to enhance transportation for all, but current research suggests that women remain less engaged and more hesitant toward their adoption. This scoping review systematically analyses 34 peer-reviewed studies published between 2016 and 2025 to assess how women’s experiences, needs, and concerns are represented in AV research. Using thematic analysis, we identify key facilitators and barriers to AV adoption and map them onto a Socio-Ecological Model spanning five levels: individual, interpersonal, institutional, community, and policy.

Our findings reveal a critical gap: while gender is frequently recorded as a demographic variable, women are rarely centred as primary users or co-creators in AV design and evaluation. Most studies rely on quantitative, mixed-gender approaches, with limited use of qualitative or participatory methods that could surface the nuanced realities of women’s mobility. As a result, vital concerns—such as safety, emotional comfort, caregiving roles, and design exclusion—are often overlooked in AV research.

To address this, we introduce the WISE-AV Framework (Women-Informed Socio-Ecological Framework), which organises the multi-level influences shaping women’s engagement with AVs. This framework extends the Socio-Ecological Model with a gender lens and integrates principles from feminist HCI to emphasise transparency, participation, and embodied experience.

Our study offers both theoretical and practical contributions: it provides a roadmap for researchers, designers, and policymakers to create more inclusive AV systems, and it delivers actionable recommendations to ensure that AVs are not just technologically advanced—but socially equitable. We argue that AVs can only fulfil their promise of safer, smarter mobility when they are designed not for a generic “user,” but with the diverse realities of women in mind.

## Introduction

The rapid development of Automated Vehicles (AVs), also known as self-driving or autonomous vehicles, promises to redefine personal and public transportation. AVs, particularly those classified by the Society of Automotive Engineers (SAE) Levels 3–5, which are enabled by artificial intelligence, sensor arrays, and real-time decision-making systems, are designed to reduce or eliminate the need for human control under specific or all driving conditions [1]. As countries and industries prepare for the mainstream adoption of these technologies, questions of equity, access, and trust become increasingly critical, especially among groups historically underrepresented in transportation research and policy, such as women.

While AVs are expected to improve traffic safety, travel efficiency, and environmental sustainability, emerging evidence suggests that their benefits may not be equitably experienced across genders [2]. Women consistently express less acceptance and greater concern about AVs compared to men. This includes reduced willingness to adopt AVs and increased apprehension about their safety and reliability [3–5]. This is further illustrated by a large-scale Australian survey of 5,089 respondents, which found that although women acknowledged the potential benefits of AVs, they were significantly less accepting and more cautious than their male counterparts [6]. However, the extent to which these perceptions are systematically investigated or meaningfully integrated into AV design and policy remains unclear.

There are compelling reasons to prioritise women in AV research. First, mobility patterns and constraints are strongly gendered. Women often travel shorter distances, make multi-stop trips (e.g., to caregiving, school, shopping), and rely more on flexible and accessible transport options [7,8]. In many contexts, women are disproportionately responsible for household logistics and caregiving, which shapes their mobility needs [7–9]. At the same time, safety and security concerns disproportionately affect women’s transport decisions, with AVs offering both opportunities (e.g., privacy, non-human drivers) and challenges (e.g., unfamiliar systems, lack of co-passenger information) [10,11].

Second, the automotive and technology sectors remain largely male-dominated, leading to design processes that may not adequately reflect the needs and concerns of women [12,13]. As a result, women may experience AVs as systems not designed with them in mind, reinforcing exclusion and mistrust [12]. Without intentional inclusion of women’s needs and concerns, AV systems risk perpetuating exclusion and reinforcing gendered inequalities in mobility access. Investigating women’s experiences is thus not only a matter of representation, but it is essential for creating safe, equitable, and trustworthy autonomous mobility systems.

To address this critical gap, we conduct a scoping review that systematically maps how women’s attitudes, perceptions, and experiences with AVs have been studied. Scoping reviews are particularly well-suited for emerging, interdisciplinary fields where concepts, methods, and definitions are still evolving [14]. Unlike systematic reviews, which typically aim to answer tightly focused questions on intervention effectiveness [15], this review aims to explore how gender has been conceptualised, identify factors influencing women’s engagement with AVs, and highlight overlooked areas for future research.

We ask: **“How has research in the field of automated vehicles focused on investigating women’s attitudes and/or gender-related disparities, and what are the factors that influence women’s perceptions and attitudes toward automated vehicles?"**

To achieve this objective, our scoping review synthesises research across disciplines, including transportation, human-computer interaction, psychology, and gender studies, with the goal of informing more inclusive AV design, policy, and practice.

## Methods

**Fig 1:**
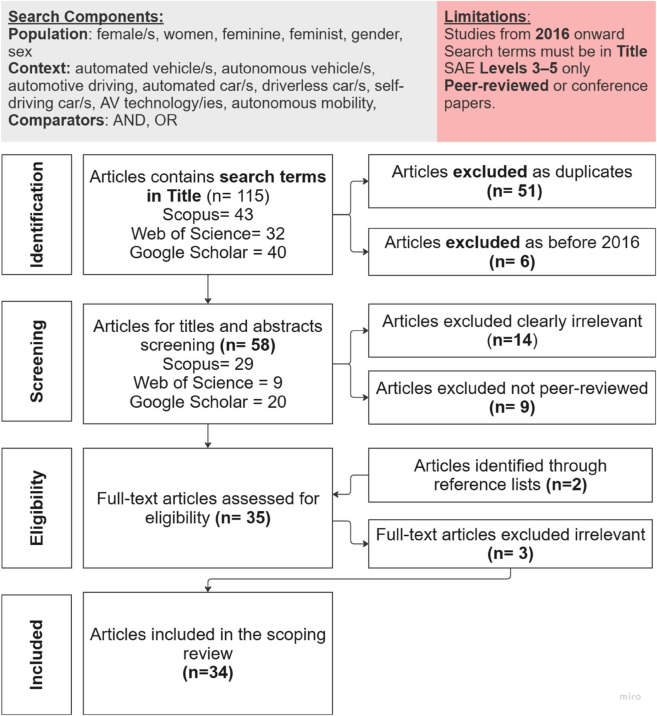
Stages of the scoping review framework: PRISMA flow diagram.

This research has been approved by Queensland University of Technology (QUT), Ethics Approval Number 5644. The scoping review followed the six-stage framework proposed by Arksey and O’Malley (2005), and later refined by Levac et al. (2010) and Tricco et al. (2018) [15–17]. This process included the systematic identification of relevant studies, application of predefined inclusion and exclusion criteria, dual screening at the title/abstract and full-text levels, and comprehensive documentation of the search strategy and selection procedures. We adhered to the PRISMA-ScR (Preferred Reporting Items for Systematic Reviews and Meta-Analyses Extension for Scoping Reviews) guidelines [17] to ensure transparency throughout the screening and inclusion process, as illustrated in Fig 1.

Although studies were not excluded based on quality, we conducted a quality appraisal using the QATSDD Assessment Tool developed by Sirriyeh et al. (2012) [18] to enhance the interpretive value of our findings. Scores ranged from 14 to 39 out of a possible 42 to 48. The appraisal was independently conducted by two authors, with a subset of studies randomly double-coded to ensure consistency and methodological rigour, see Sect Quality appraisal for details.

For analysis, we employed Braun and Clarke’s six-phase framework for thematic analysis [19], which is widely applied in qualitative and scoping review research. Two researchers independently coded each study across five high-level analytical themes that emerged from the studies. To assess inter-coder reliability, we calculated Cohen’s Kappa κ for each domain, with values ranging from 0.71 to 0.96, indicating substantial to almost perfect agreement based on Landis and Koch’s (1977) interpretive benchmarks [20]. Discrepancies were resolved through discussion to reach full consensus prior to synthesis.

The following subsections outline the six stages of our scoping review in detail.

### Search strategy

An initial broad search strategy returned a high volume of irrelevant studies. To improve precision and ensure alignment with the review’s gender-focused objective, we refined our strategy based on the PCC framework (population, concept, and context) recommended as a guide by the Joanna Briggs Institute (JBI) for developing eligibility criteria and guiding inclusion/exclusion decisions in scoping reviews [21], and restricted search terms to the article title field. This targeted strategy prioritised studies that explicitly centred on women, gender, or feminist perspectives within the context of AVs, as described in Table 1.

**Table 1:** Search components for AV-related studies from gender perspective.

Criterion	Details
Population terms	female/s, women, feminist, gender, sex
Concept	Attitudes, perceptions, concerns, or behavioural intentions regarding AVs
Context terms	automated vehicle/s, autonomous vehicle/s, automotive driving, automated car/s, driverless car/s, self-driving car/s, AV technology/ies, autonomous mobility

We conducted our search across three major interdisciplinary databases: Scopus, Web of Science, and Google Scholar. These platforms were selected due to their extensive coverage of peer-reviewed literature across the fields of engineering, transport studies, social science, and human-computer interaction, ensuring a balanced representation of both technical and behavioural research relevant to AVs.

The search employed Boolean logic (AND/OR) to combine population terms (female/s, women, feminist, gender, sex) with context terms (automated vehicle/s, autonomous vehicle/s, automotive driving, automated car/s, driverless car/s, self-driving car/s, AV technology/ies, autonomous mobility). These terms were iteratively tested and refined to maximise the relevance of retrieved studies.

The literature search was conducted in two phases to ensure the review captured the most up-to-date evidence. The first search, led by the first author, was conducted between September and December 2023 and covered studies published from January 2016 to December 2023. A follow-up search was conducted in April 2025 by the second author, using the same databases, search terms, and inclusion criteria as the initial search. This update was designed to ensure that the review reflects current developments in the rapidly evolving field of AV research. Both reviewers worked independently, and results were later merged and screened collaboratively.

### Study selection

We conducted our study selection in two phases, corresponding to our two search periods. We first removed the duplicates; then we screened all remaining records by title and abstract to check for relevance. Before starting this process, we (the first and second authors) held several consensus meetings to agree on the inclusion and exclusion criteria, which we then applied consistently throughout the review. In both the initial and updated searches, we independently reviewed the titles and abstracts. When we disagreed, we discussed the differences and reached a consensus together. For studies that seemed potentially relevant, we retrieved the full texts and assessed them based on the agreed eligibility criteria. Our decisions were guided by a shared understanding of the research question and the focus of this review. This helped ensure that all included studies aligned with the purpose of our work.

#### Relevant studies.

To ensure alignment with the research objectives, we outlined the inclusion and exclusion criteria in Table 2. Studies were included in the review if they: (1) were peer-reviewed empirical or theoretical articles, (2) focused explicitly on gender or women’s perceptions, adoption, or interaction with AVs, (3) involved human participants, either as drivers or passengers, (4) studies involving mixed-gender samples were eligible, provided that gender was used as a key analytic category in the findings. (5) were published between 2016 and April 2025. We chose this period to align with the formal adoption period of SAE’s AV classification framework [1,22] and the emergence of more applied, user-focused AV studies. Prior to 2016, literature lacked consistent definitions of automation and often discussed AVs in abstract or conceptual terms. (6) Finally, only studies published in English were included. We limited our inclusion to English-language full-text studies due to resource constraints and concerns about translation accuracy, particularly regarding nuanced methodological or cultural terms related to AV perception. We chose not to use online translation tools, as the semantic precision required for our thematic synthesis could be compromised [23]. However, future work should consider multilingual inclusion using translation tools. In contrast, studies were excluded if they were not peer-reviewed, focused exclusively on SAE levels 0–2, which involve human-dominated control and do not represent true automation, making them outside the scope of our focus on fully autonomous adoption and gendered user interaction. Additionally, studies that investigated AV interactions limited to pedestrians, or did not present gender-disaggregated results, or did not consider gender as a core variable in the analysis were also excluded from the review.

**Table 2:** Inclusion and exclusion criteria for study selection.

Criteria	Inclusion Criteria	Exclusion Criteria
Study Type	Primary empirical research (quantitative, qualitative, or Mixed-methods)	Review articles, conference abstracts, book chapters, commentaries, and editorials
Population	Human participants identifying as women, or studies that analyze gender as a key variable. Gender was defined as being a biological male or female	Studies that do not include a gendered lens or do not report disaggregated gender findings
AV Level	Studies involving SAE level 3 to 5 automated vehicles	Studies focused solely on SAE levels 0 to 2
Focus Area	Studies examining attitudes or experience with AVs	Studies focused only on technical, engineering, or traffic modelling aspects without user perception data
Study Design	Peer-reviewed studies employing quantitative, qualitative, or Mixed-method designs	Non-peer-reviewed materials or anecdotal opinion pieces
Time Period	Studies published between 1 January 2016 and 10th April 2025	Studies published before 2016
Language	Full-text available in English	Articles in other languages without reliable English translation or access to full text
Accessibility	Full-text accessible through academic databases	Studies available only as abstracts, or full-text articles that cannot be accessed due to paywall restrictions

#### Quality appraisal.

Two researchers independently conducted a quality assessment following the QATSDD framework [15] to evaluate the methodological rigour of all included studies. Initially, both authors jointly assessed four randomly selected studies to ensure consistency in interpretation and scoring. Following this calibration, the first reviewer independently appraised 20 studies, and the second reviewer evaluated the remaining 10 studies. The reviewers then discussed discrepancies until agreement.

While we report QATSDD scores (ranging from 14 to 39) to illustrate variation in reporting quality, no studies were excluded based on quality, in alignment with scoping review guidance [24]. Our aim was to map the existing literature broadly rather than restrictively evaluate only high-quality studies. Our rationale follows Grant and Booth’s typology for scoping reviews, which emphasises comprehensiveness and inclusivity over formal exclusion based on quality [24]. The scoring outcomes are presented in Table 3.

**Table 3:** Characteristics of included studies.

Ref	Year	Area	AV Level	Sample			Age Group	Method	Data Source	QATSDD score
				size	Female No.	Gender composition				
[26]	2021	Saudi Arabia	L4 AVs	1400	1400	Female-only	Mixed-age	Quantitative	Survey-based	32
[27]	2018	USA	L4 AVs	441	254	Mixed-gender	Mixed-age	Quantitative	Survey-based	31
[28]	2023	China	AVs	1082	638	Mixed-gender	Mixed-age	Quantitative	Survey-based	34
[29]	2022	USA	L3 AVs	404	221	Mixed-gender	Mixed-age	Quantitative	Survey-based	32
[30]	2023	USA	L3 AVs	28	14	Mixed-gender	Mixed-age	Quantitative	Simulation-based	35
[8]	2022	Germany	AVs	31	31	Female-only	Mixed-age	Mixed-method	Focus groups + surveys	39
[31]	2018	USA	AVs	1335	774	Mixed-gender	Mixed-age	Quantitative	Survey-based	24
[32]	2019	Czech	L4 AVs	1065	522	Mixed-gender	Mixed-age	Mixed-method	Focus groups + surveys	36
[33]	2023	Hungary	AVs	1273	490	Mixed-gender	Mixed-age	Quantitative	Survey-based	34
[34]	2016	Germany	AVs	1603	848	Mixed-gender	Mixed-age	Quantitative	Survey-based	32
[35]	2018	UK	AVs	925	324	Mixed-gender	Mixed-age	Quantitative	Survey-based	28
[3]	2019	USA	AVs	158	114	Mixed-gender	Mixed-age	Quantitative	Satistical modeling	24
[5]	2022	UK	L3 AVs	76	33	Mixed-gender	Mixed-age	Mixed-method	Simulation-based + surveys	23
[36]	2022	China	L3 AVs	301	134	Mixed-gender	Mixed-age	Quantitative	Survey-based	31
[37]	2019	USA	AVs	60	31	Mixed-gender	Mixed-age	Mixed-method	Simulation-based + surveys	27
[38]	2022	Hungary	AVs	496	223	Mixed-gender	Mixed-age	Mixed-method	Simulation-based + surveys	19
[39]	2022	Poland	AVs	227	148	Mixed-gender	Young adults	Quantitative	Survey-based	21
[40]	2021	Some Europe countries	SAVs	1772	556	Mixed-gender	Mixed-age	Quantitative	Survey-based	16
[11]	2022	Germany	SAVs	21	21	Female-only	Mixed-age	Qualitative	Focus groups	14
[12]	2021	Germany	SAVs	11	6	Mixed-gender	Mixed-age	Qualitative	Semi-structured interviews	22
[41]	2023	USA	L4 AVs	101	56	Mixed-gender	Mixed-age	Quantitative	Survey-based	28
[42]	2022	Spain	AVs	856	423	Mixed-gender	Mixed-age	Quantitative	Survey-based	30
[43]	2022	USA	AVs	525	220	Mixed-gender	Not reported	Quantitative	Survey-based	34
[4]	2022	Germany,USA	AVs	715	382	Mixed-gender	Mixed-age	Quantitative	Survey-based	15
[2]	2022	Germany	L3 and L5 AVs	725	351	Mixed-gender	Mixed-age	Mixed-method	Simulation + Surveys + open-ended questions	14
[44]	2022	USA	SAVs	413	200	Mixed-gender	Mixed-age	Quantitative	Survey-based	30
[45]	2023	Germany	AVs	726	351	Mixed-gender	Mixed-age	Quantitative	Survey-based	14
[46]	2016	Germany	AVs	48	27	Mixed-gender	Young adults	Mixed-method	xSimulation-based + ECG recordings	14
[47]	2024	Bangladesh	SAVs	23	23	Female-only	Mixed-age	Mixed-method	Simulation-based + surveys	22
[13]	2024	Netherlands	AVs	23	23	Female-only	Mixed-age	Mixed-method	Focus groups + in-depth interviews	26
[48]	2024	Japan	L3 AVs	30	15	Mixed-gender	Mixed-age	Mixed-method	Simulation-based + surveys	33
[49,50]	2024	Some Europe countries	L3 AVs	8412	4065	Mixed-gender	Mixed-age	Quantitative	Survey-based	19, 21
[51]	2025	USA	AVs	1881	1046	Mixed-gender	Mixed-age	Quantitative	Survey-based	25

### Data charting and thematic synthesis

We followed the six phases of Braun and Clarke’s inductive approach, allowing themes to emerge organically from the data as follows:

**(1) Familiarisation with the data:** Both reviewers independently read and re-read all included studies to become familiar with the key content areas. We charted and analysed a range of descriptive variables from each study to build a contextual understanding of the dataset. This included information such as publication year, geographic location, AV type and level, participant gender and age, and methodological design. These characteristics were detailed in Table 3:

1. **Gender sample composition:** Studies were categorised as “Female-only” for studies that focus on women as the sole participant group, or “Mixed-gender” for studies that use both male and female participants.
2. **Age groups:** Studies were grouped as “young adults” (18–26 years), “older adults” (65+), “mixed-age” (27–64), and “not reported” if no age info was given.
3. **Research methods:** Studies were coded as “qualitative”, “quantitative”, or “mixed methods” based on their design and use of techniques such as surveys, interviews, simulations, driving tests, or physiological tracking (e.g., ECG).
  - Studies using semi-structured interviews, focus groups, or thematic analysis of participant narratives were coded as **qualitative**.
  - Those using structured surveys, simulation trials, or statistical modelling were coded as **quantitative**.
  - Studies combining multiple approaches (e.g., interviews + driving simulation, or surveys + open-ended questions) were categorised as **Mixed-methods**.
4. **AV type and level:** We categorised studies according to the SAE levels referenced: L3, L4, L5, and the specific AV type (e.g., shared automated vehicles/SAVs, connected automated vehicles/CAVs, or fully automated vehicles/AVs). When automation levels were unspecified but described as full autonomy, they were treated as L5/AVs [1].

**(2) Generating initial codes:** In this phase, we began identifying recurring content and coding segments related to women’s perceptions, trust, safety concerns, and usage patterns. These early codes were grounded in both the descriptive metadata and substantive findings from each study. **(3) Reviewing study focus and generating codes:** Using the extracted data, we began by identifying patterns in the focus of the included studies. Based on the content of the findings and discussion sections, we defined thematic categories of study focus, and to ensure reliability in thematic coding, we calculated Cohen’s Kappa (κ for 20 out of the 34 included studies to assess the level of agreement across key focus areas and cross-cutting themes. The agreement between the two reviewers was very high: **Overall thematic focus area categories (5 themes)**: κ = 0.96, P0 = 0.972, P*e* = 0.314. Table 4 describes the five primary study focus categories.

**Table 4:** Definitions of the five thematic focus area categories used in the review.

Theme	Definition	Examples
**Acceptance and Adoption**	Studies that explore the willingness to use, intention to adopt, or general attitudes toward AV technology	Behavioural intention, technology acceptance models, preference for human vs. autonomous control
**Prevalence of AV Knowledge**	Studies that assess participants’ familiarity, prior experience, or knowledge of AV systems or their preferred source of awareness regarding AVs	Exposure to AV demos, awareness of SAE levels, perceived understanding of AV functions
**Safety and Security Aspects**	Studies focused on perceptions or concerns related to physical, emotional, or data-related safety in AV contexts	Fear of harassment, perceived crash risk, trust in AV decision-making, concerns about ride-sharing safety
**Takeover Performance**	Studies evaluating how drivers’ gender impacts human performance during system disengagement	Reaction time, takeover quality, age- or gender-based comparisons in performance, simulator-based tasks
**Focus on Women’s Perspectives**	Studies that centre women as a distinct user group or provide disaggregated gender analysis in AV research.	Mobility patterns of women, caregiving-related transport needs, gendered technology design or critique

**(4) Generating and grouping thematic codes:** From this process, we conducted a detailed reading of the findings and discussions to extract what female participants reported or how gender differences were interpreted within each study. We developed an initial list of emerging themes grounded in what women reported as influencing their perceptions of AVs. As these themes began to recur across multiple studies, we systematically grouped them into broader, higher-order categories, reflecting more consistent patterns of experience. These grouped themes were then conceptually organised into two overarching domains: facilitators (factors that enabled positive perceptions or adoption) and barriers (factors that hindered or constrained AV use).

**(5) Defining and naming themes:** Then the two reviewers collaboratively synthesised the findings by grouping the identified codes (barriers and facilitators) into higher-order thematic categories. Each reviewer independently reviewed study findings and coded the presence or absence of themes using a pre-defined set of five overarching thematic domains: (1) Trust and Perceived Safety/Security, (2) Exposure, Familiarity, and Proficiency with Technology, (3) Equity Access, Policy Support, and Social/Cultural Norms, and (4) Accessibility, Usability, and Interaction Design. To assess the level of agreement between reviewers, we calculated Cohen’s Kappa (κ) for each thematic domain using contingency tables of presence/absence labels. The agreement across themes was substantial to near-perfect, with Kappa scores ranging from κ=0.705 to κ=0.8. Specifically, the “Equity Access, Policy Support, and Social/Cultural Norms" theme achieved the highest agreement (κ=0.8), while “Trust and Perceived Safety/Security" and “Accessibility, Usability, and Interaction Design" both yielded κ=0.76. For “Exposure, Familiarity, and Proficiency with Technology," inter-rater reliability was substantial, κ=0.705. This reflects substantial agreement. All Kappa values exceeded 0.70, demonstrating substantial to near-perfect agreement [20]. All disagreements were subsequently resolved through consensus meetings.

**(6) Synthesising and Reporting Findings:** Finally, final codes were integrated in light of the Socio-Ecological Model (SEM) [25], enabling a layered analysis that distinguished between individual, interpersonal, institutional, community, and policy-level influences on women’s AV perceptions.

## Results

Our review included 34 peer-reviewed studies published between 2016 and April 2025. We have used pivot tables and charts to illustrate the frequency and extent of women’s inclusion in AV research. Our results indicate that while gender is increasingly being considered in AV research, women are not consistently centred as primary users or informants. Most studies use mixed-gender samples without tailoring their research designs to women’s mobility needs, safety concerns, or caregiving roles. Furthermore, methodological and geographic gaps persist, particularly in the Global South and among aging or low-income populations. The limited integration of participatory and feminist approaches further hinders the field’s ability to generate gender-equitable innovation in autonomous transportation.

### Geographical distribution of the studies

**Fig 2:**
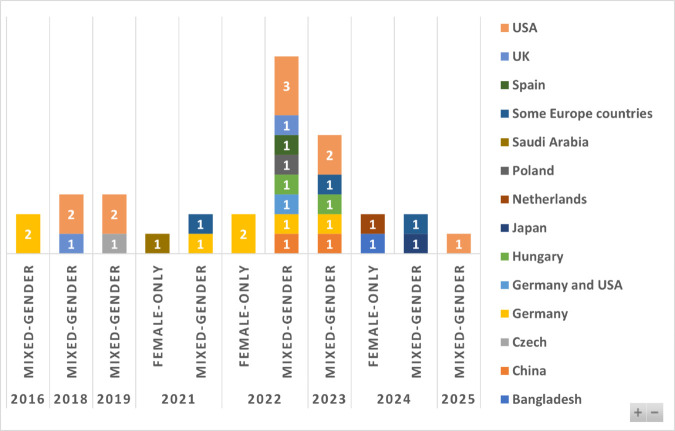
Distribution of included AV studies by gender composition, publication years and countries.

Fig 2 shows the geographical distribution of the included AV studies by gender composition of participants (Female-Only vs. Mixed-Gender) and country of study, from 2016 to 2025. Mixed-gender studies dominate across the period, representing the vast majority of AV research. Only five female-only studies were identified over the entire decade, accounting for just 15% of the total sample. The most prolific publishing years were 2022 and 2023, with 10 and 6 studies, respectively. Female-only studies were absent before 2021 and appeared only sporadically in 2021, 2022, and 2024.

Geographically, research is concentrated in high-income Western countries. The USA leads in AV gender-focused research, particularly in Mixed-Gender studies, with notable peaks in 2018, 2019, and 2022. Germany and joint Germany–USA studies appear in both gender categories, suggesting some sustained attention to gender perspectives in these contexts. A few Global South countries (e.g., Saudi Arabia, Bangladesh) are represented, mainly in Female-Only studies—Saudi Arabia in 2021 and Bangladesh in 2024. European contributions include Poland, Hungary, and the Netherlands, as well as multi-country projects in Southern Europe, though these are predominantly Mixed-Gender.

In total, the chart reveals a clear gender imbalance in AV research participation, with Mixed-Gender samples dominating and relatively few studies centred on women’s perspectives. Global representation is also uneven, with North America and Western Europe leading the field. This geographic skew underscores an important limitation in the current evidence base: perspectives from women in lower-income or underrepresented regions remain scarce. Given that AV adoption and perceptions are likely to vary across cultural, infrastructural, and socioeconomic contexts, this imbalance highlights a gap in both equity and representation in the global AV research landscape.

### Gender and age composition of study samples

**Fig 3:**
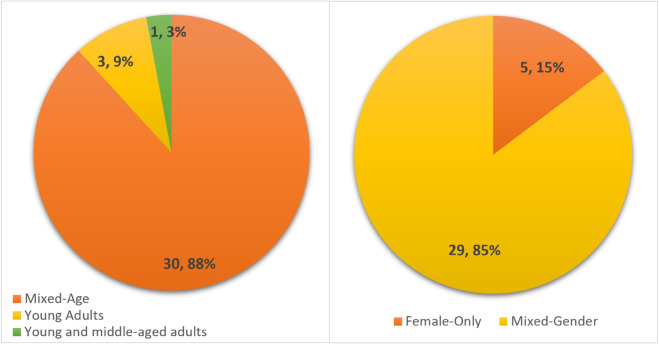
Distribution of included AV studies by gender composition and age.

Fig 3 presents the gender and age distribution of participants across the 34 studies included in this review. The pie chart on the left shows that the overwhelming majority of studies (n = 29; 85%) employed mixed-gender samples. Only 5 studies (15%) focused exclusively on women participants. This indicates a major limitation in the literature: while many studies include women as part of their sample, few are designed specifically to centre women’s voices, preferences, or mobility needs in relation to AVs.

The pie chart on the right highlights that 88% of the studies (n = 30) involved participants from mixed-age groups, which typically included adults of varying ages, from early adulthood to older adulthood. Only a small number of studies focused on narrower age ranges, such as young adults (n = 3; 9%) or young and middle-aged adults (n = 1; 3%).

These patterns show that, although gender and age are often recorded, they are rarely the main focus of analysis. This highlights the value of our review in identifying where gender—especially women’s experiences—is not just mentioned but examined in depth in AV research. It also points to the need for more targeted studies on how women in different age groups experience AVs, particularly older adults who may face unique challenges with accessibility, familiarity, and trust.

### AV types and SAE levels with gender composition

**Fig 4:**
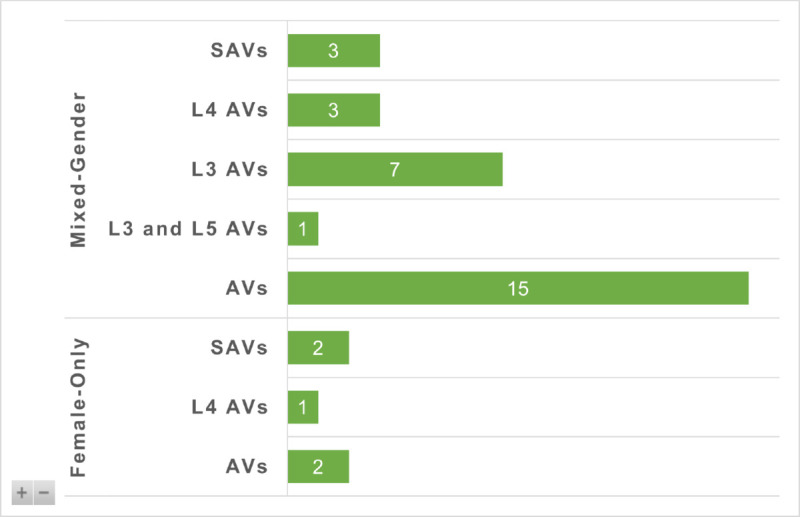
AV types and SAE levels of AVs across studies based on gender composition.

Fig 4 illustrates how different types and levels of AVs are represented across studies by gender composition—female-only versus mixed-gender samples.

Among the 29 mixed-gender studies, the majority (n = 15) examined AVs without specifying a particular SAE automation level, often using generic or fully autonomous scenarios. Seven focused on Level 3 (L3) AVs, which involve conditional automation and allow researchers to study trust, control, and takeover behaviour. Three studies explored Level 4 (L4) AVs, and only one compared both Level 3 and Level 5 (L5) AVs. Notably, three studies in this group addressed Shared Automated Vehicles (SAVs), reflecting growing interest in ride-hailing and public-use contexts.

In contrast, the five female-only studies show a narrower but more targeted focus. Two examined general AVs, two focused on SAVs, and one investigated L4 AVs. None addressed L3 or L5 AVs, and none compared multiple SAE levels.

These findings reveal two main gaps. First, mixed-gender research is concentrated at the abstract or fully autonomous end of the spectrum, with fewer studies engaging with specific SAE levels that reflect current real-world capabilities. Second, female-only studies remain rare and unevenly distributed across AV types. The absence of gender-specific research on L3 AVs is particularly concerning, as this level is currently the most prevalent in testing and regulatory planning.

### Study design and data collection methods

**Fig 5:**
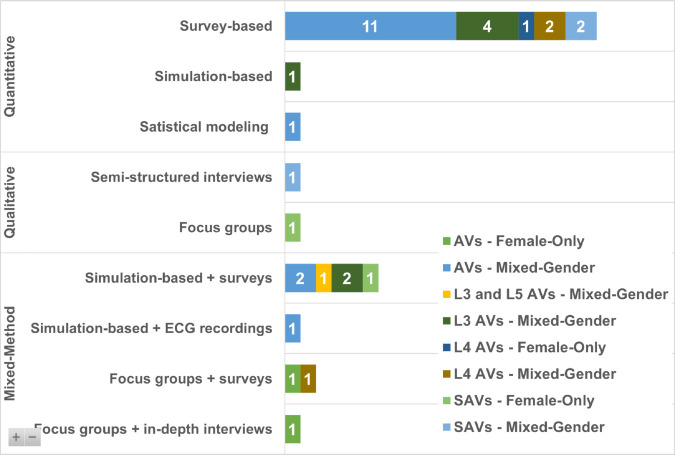
Research methods and data sources used in reviewed publications.

Fig 5 presents the methodological breakdown of the 34 studies included in this scoping review, highlighting their research design (quantitative, qualitative, or mixed-method) and the data collection techniques employed.

The majority of studies (n = 22) employed a quantitative approach, with most relying on survey-based data collection (n = 20). This highlights the field’s predominant emphasis on measuring attitudes, preferences, and acceptance patterns through large-scale questionnaires targeting mixed-gender populations. While these studies offer valuable insights into general public sentiment across diverse global regions, they typically address gender only as a demographic variable, rather than centring women’s specific experiences or concerns. Notably, none of these studies were designed with female-only samples.

Only a small subset of quantitative research used simulation environments (n = 1) or statistical modelling techniques (n = 1), indicating minimal integration of behavioural realism or context-specific dynamics. This presents a clear gap: future research should prioritise women-only studies.

Only two studies adopted a purely qualitative approach, one using semi-structured interviews with mixed-gender participants and another relying on focus groups with female-only participants, which enabled a deeper understanding of women’s experiences with AVs; however, qualitative methods remain underutilised relative to the topic’s complexity.

Mixed-methods approaches were employed in 10 studies, reflecting a growing recognition of hybrid designs that offer richer insights. The most common combinations included simulation-based and survey-based designs (n = 6), followed by focus groups with surveys (n = 2) and in-depth interviews (n = 1), and one study that used ECG recordings alongside simulator tests.

While the mixed-method trend is promising, it is notable that few studies triangulated their findings across methods and only two focus on female-only perspectives, pointing to a persistent gap in evidence about how women’s perceptions evolve over time and with direct AV exposure.

These findings underscore the importance of expanding qualitative future AV research, particularly to better represent underexplored female voices and contextual influences.

### Women’s inclusion by thematic focus and AV type

**Fig 6:**
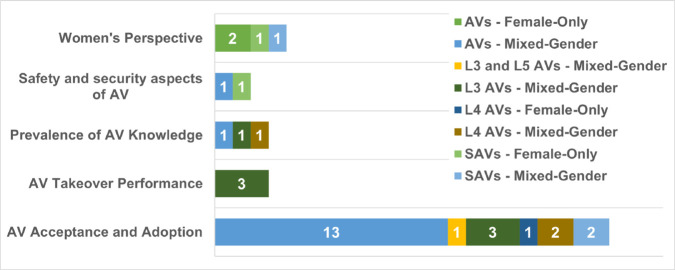
Distribution of included AV studies by thematic focus, AV type, and gender composition.

Fig 6 presents the thematic breakdown of the 34 studies included in this scoping review, showing their research focus, AV type, and participant gender composition. This layered view reveals not only which topics dominate AV literature but also how AV type and gender inclusion shape the research lens.

The vast majority of studies on *AV Acceptance and Adoption* used mixed-gender samples and a generalised AV type (n = 13). This pattern reflects a strong emphasis on broad adoption modelling rather than on nuanced, demographic-specific exploration. Only a few of these studies examined specific AV types such as L3, L4, or SAVs in a disaggregated way, potentially missing the contextual detail that different AV designs bring to the user experience.

In contrast, research explicitly focused on *Women’s Perspectives* was rare (n = 4), with only two studies using female-only participants. These studies, however, covered a more varied set of AV types—including SAVs and general AVs—indicating that gender-targeted work can yield richer, context-sensitive insights.

Safety and Security Aspects appeared in only three studies: one on SAVs, one on AVs, and one on L3/L5 AVs. Of these, only one focused exclusively on women. Given the centrality of safety concerns in shaping women’s attitudes toward AVs, this underrepresentation signals a critical research gap.

*Prevalence of AV Knowledge* was similarly underexplored. The three studies on this theme covered L4 AVs and general AVs, with both mixed and female-only samples. While these studies highlight the relevance of awareness and information preferences, they remain peripheral to the broader research agenda.

The only theme where AV type was closely matched to use-case was *AV Takeover Performance*. All three studies in this category examined L3 AVs with mixed-gender samples—logical given that L3 autonomy involves conditional handover scenarios. However, none explored women-specific reactions or stress responses, revealing a gender data gap in performance-critical contexts.

This distribution underscores a broader finding: most AV research treats gender as a comparative variable rather than as a central lens for design or engagement. There is an urgent need for more studies combining female-only samples with specific AV types—especially SAVs and L4/L5—and moving beyond adoption metrics to address trust, safety, emotional comfort, and design equity.

### Study themes by methodology and sample composition

**Fig 7:**
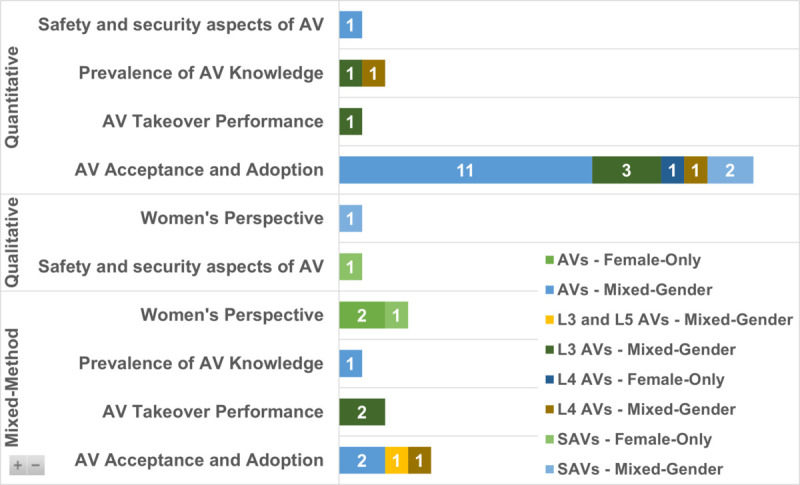
Distribution of included AV studies by thematic focus areas across methodological approaches, with types of AVs and gender composition.

Fig 7 summarises the thematic focus areas of the reviewed studies, organised by methodological approach, AV type, and gender composition. This multidimensional view shows how AV research incorporates—or overlooks—gender considerations across different research lenses.

The most common approach is quantitative, especially in the “AV Acceptance and Adoption" category, with 17 mixed-gender studies examining general perceptions, willingness to adopt, and influencing factors such as age, trust, and experience. Only one quantitative study used a female-only sample, revealing a major gender representation gap.

Qualitative and mixed-method studies centred on Women’s Perspectives remain rare—just one qualitative study (mixed-gender) and three mixed-method studies (female-only). This is notable, as these methods are well-suited to capturing rich, contextual, and experiential insights that can inform inclusive design.

Safety and security are addressed in only two studies: one quantitative (mixed-gender) and one qualitative (female-only). AV knowledge appears in just three studies, each using different methodologies and populations, suggesting limited exploration of how women access information or understand AV technology.

AV Takeover Performance—critical for conditional automation (e.g., L3)—was examined only in female-only, mixed-method studies, likely due to the suitability of simulation-based testing for this topic.

Across all themes, mixed-gender samples dominate, and Women’s perspectives were the central focus in only four studies: three mixed-method and one qualitative. This highlights a substantial underrepresentation, despite the clear importance of gendered experiences in mobility and automation.

## Findings

Our review uncovered a wide and multi-layered set of factors that either support or hinder women’s engagement with AVs, based on 34 peer-reviewed studies published between 2016 and 2025. Facilitators include features that build trust, enhance usability, or encourage adoption, while barriers encompass factors that create hesitation, raise safety concerns, or reduce access and confidence.

To organise these findings, we applied the Socio-Ecological Model (SEM) [25], which helped us understand how various types of influence operate across five levels: individual, interpersonal, institutional, community, and policy. SEM not only identifies where influences occur but also provides a framework to categorise the nature of these influences and the types of interventions required at each level [52].

Tables 5 and 6 summarise the key facilitators and barriers, along with the studies that reported them. For instance, emotional comfort and familiarity with technology were mapped at the individual level, while peer influence and social norms were placed at the interpersonal level. Design features and interface usability were associated with the institutional level, affordability and rural access were categorised under the community level, and regulatory issues, legislation, and public trust fell under the policy level.

**Table 5:** Facilitators influencing women’s attitudes toward AVs, categorised by SEM levels.

Code	Definition	Facilitators (Supporting Studies)	SEM Level
**Technology familiarity and exposure**	Encompasses users’ prior exposure to AVs or related technologies (e.g., smart homes, ADAS), as well as their access to clear, real-time, and engaging information that builds trust and supports informed interaction.	Experiences with ICT/ADAS [26,53]; Prior hands-on experience [32,33,41]; Driving experience [5,48]; AV familiarity [5,27,37,37,38,41,43,53]; Real-time info [11,13]	Individual
**Emotional and psychological comfort**	Personal innovativeness and enjoyment tied to stress relief, entertainment, and satisfaction during AV travel, including positive experiences with NDRTs.	Emotional stability [13,27]; Hedonic motivation [26,28,38,46,48,50]; Willing to try [26,27,36]	Individual
**Perceived safety and security**	Perceptions of safety, efficiency, and comfort, including features that prevent harm and provide reassurance during AV use.	In-vehicle safety features [3,8,12,26,35,42,44,47,51]; Driver absence as safety [11,13]	Individual
**Social influence and norms**	Positive influence from peers, family, or broader social support systems encouraging AV adoption.	Subjective norms (social support) [26,36]	Interpersonal
**User-centred design and representation**	Inclusion of women’s needs in AV design, such as comfort, cultural sensitivity, and interface personalisation.	Design preferences [44,48]; Tailored content [38,44]; Comfort and layout [26,44,46]	Institutional
**Sustainability and equity**	Environmental and societal values met through AV use, including reduced emissions and inclusive access, especially for underserved or non-driving populations.	Greener transport[8,45]; Inclusive access [8,13,45,51]	Community
**Supportive policy and governance**	Government policies, legal frameworks, and official endorsements that enhance trust and legitimise AV adoption.	Regulatory support [8]	Policy

**Table 6:** Barriers influencing women’s AV adoption, organised by SEM level.

Code	Definition	Barriers (Supporting Studies)	SEM Level
Lack of familiarity and tech proficiency	Barriers related to awareness, familiarity, and usage experience with AVs and related technologies.	Lack of AV knowledge [32,35,43]; Tech savviness [33,50,54]; Info sources [32]; Complexity of AVs [2,3,32,35]	Individual
Emotional discomfort and psychological sensitivity	Presence of fear, anxiety, tension, or emotional distress related to AV use, especially among individuals with less confidence or higher psychological sensitivity.	Emotional response [3,33,34,38,44,46,51]; Personality traits [27]; Low performance satisfaction [28,46]; Willingness to pay [2,53]	Individual
Safety and security concerns	Fears related to physical safety, absence of human drivers, or unclear prioritisation of user safety in AV decision-making.	Safety concerns [8,11,12,44,47–49]; Privacy concerns [8,11–13,47]	Individual
Trust and desire for control	Distrust in AV technologies and heightened perception of operational or data-related risks. Includes discomfort with the absence of manual driving control or the inability to override AV behaviour.	Technical distrust [3,8,13,30,35,44,45,47,50,51]; Desire for control [4,8,11,12,27,46,47]; Cautious behavior [5,30,35,48]	Individual
Demographic influences on adoption	Barriers linked to age, gender, education, parenting, or location.	Age-related gaps [2,4,27,32,34,35,37,41,42,49,51,53]; Lower education [43,51]; Low travel demand [2,8]; Parenting status [31]; Gendered performance gaps [5,37,48]	Individual
Gender and social norm constraints	Barriers stemming from gender norms, scepticism from peers, conservative or religious expectations, or mixed-gender co-passenger concerns.	Solo ride discomfort [11,13,36,40,47]; Peer pressure [12,26,28,36]; Commonalities concerns [13,33]; Cultural influence [35,39,40,47]	Interpersonal
Design and layout dissatisfaction	When AVs fail to consider gendered needs or caregiving roles, leading to discomfort. Includes concerns about hygiene, unexpected interior features, or discomfort inside AVs.	Lack of access [8]; No women-only options [11,12]; Gendered design absence [13,47,48]; Layout issues [3,48]; Cleanliness [11,13]; Rating concerns [11]	Institutional
Socio-cultural and environmental context	Structural barriers at the community level, including infrastructure, cost, and flexible service limitations.	High costs [28,38,40,43]; Stereotyping [13]; Rural living [8]; Travel time inefficiency [8,11,26]; Sustainability concerns [45]; Cultural influence [35,39,40,47]	Community
Legal and ethical uncertainty	Concerns over the legal status, ethical logic in decision-making, and unclear data/privacy governance.	Ethical/legal ambiguity and policy gaps [8]	Policy

### Individual-level influences

At the individual level, the review revealed that both barriers and facilitators were the most frequently cited influences across the reviewed studies. Although most of the 34 reviewed studies employed survey-based, quantitative methods with mixed-gender samples, few specifically explored gendered emotional responses to AV technology. The following points explain the individual-level factors influencing AV adoption:

1. **Emotional and psychological comfort or discomfort:** Women’s adoption of AVs is strongly influenced by emotional and psychological factors, particularly when parenting responsibilities are involved. Studies have shown that when AV travel is perceived as enjoyable or relaxing, acceptance increases [26,28,48]. Many women value the chance to use in-vehicle time for non-driving-related tasks (NDRTs), such as reading, working, or resting—which can relieve multitasking and caregiving stress [50]. This emotional comfort is especially appreciated by mothers, for whom commuting time can serve as a rare personal space [51].
  However, these potential benefits are offset by anxiety in solo or shared AV rides: fear, perceived vulnerability, and the absence of human supervision are recurrent barriers[3,27,44,45,49]. These concerns are amplified for parents, where child safety heightens perceived risk. Mothers have been found to be more hesitant than fathers to allow children to travel alone in AVs [31].
2. **Technology familiarity and exposure:** Another recurring theme was that prior exposure to technologies such as smart systems, ADAS, or simulator trials was consistently associated with higher AV acceptance [26,33,53]. Studies noted that when women had hands-on experience or access to clear, real-time information about AV operation, they showed greater willingness to engage with the technology and reported lower levels of uncertainty or fear [26,33,41,45,53].
  In contrast, a lack of familiarity and technological proficiency—especially among older women or those with limited digital exposure—was associated with lower acceptance [32,53]. This demographic was underrepresented in many studies; however, when included, results showed a clear association between lower exposure and reduced confidence or interest in AV use. This underscores the importance of digital literacy and targeted onboarding in AV adoption strategies.
3. **Trust, Control, and Perceived Safety:** Trust and the ability to retain some control are central to acceptance. Many women expressed scepticism toward the reliability of AV systems, particularly in situations where manual override was unavailable. AVs are expected to be designed for safety and predictability, aligning with the cautious and reflexive safety responses often observed among women [28,48].
  Studies found that perceptions of safety linked to features like surveillance systems, secure routing, and the absence of male drivers improved women’s confidence in using AVs [3,11,13,44,47]. However, trust was often undermined by concerns about losing control, being unable to override vehicle behaviour, and doubts about the system’s ability to handle emergencies [3,8,13,30,35,36,46,47].
  This tension between the desire for enhanced safety and the need for control reflects a deeper psychological conflict. Many women reported that even when AVs included advanced safety features, they felt anxious without a human driver present or a clear way to intervene [11,12]. The inability to intervene heightened feelings of vulnerability, reducing both perceived safety and trust—core influences on adoption intent.
4. **Demographic factors:** Age, education, caregiving status, and place of residence shape women’s engagement with AVs. Women with caregiving responsibilities value the possibility of repurposing in-vehicle time (e.g., to rest or complete tasks), but they also worry about children’s safety—especially on school trips or when kids travel alone [31,51]. In rural areas, distances, sparse services, and limited infrastructure further make AVs seem less useful [8,31].

These four points above illustrate how emotional, technological, and demographic factors interact to influence AV adoption among women.

### Interpersonal-level influences

Although interpersonal forces are less examined than individual factors, peer influence, family expectations, and cultural norms can shape women’s AV adoption.

Social influence and peer support emerged as an important facilitator. When women received encouragement or approval from peers, family members, or broader social networks, they were more likely to express interest in using AVs [26,36]. This reflects the importance of subjective norms and social reinforcement, as identified in several studies where peer endorsement positively correlated with adoption intent.

Conversely, many interpersonal barriers were rooted in gendered social norms and constraints. Scepticism or disapproval from close social contacts—especially in conservative or traditional cultural contexts—often led to reluctance. These barriers were amplified by discomfort with mixed-gender co-passengers, concerns about solo travel, and the broader cultural expectations surrounding women’s mobility [11,13,36,40,47]. Peer pressure and social scrutiny were also reported as deterrents, particularly among women who feared judgment for embracing new technologies [12,28,33].

Additionally, cultural and religious norms shaped how women perceived the appropriateness and safety of AV travel. In certain contexts, traditional gender roles and expectations constrain autonomy in public or shared mobility, influencing both actual behaviour and stated willingness to adopt AVs [35,39].

### Institutional-level influences

This level relates to how AV systems are designed, developed, and presented by manufacturers, designers, and service providers. Across the reviewed studies, participants’ experiences with user interfaces, cabin layout, and design responsiveness had a strong impact on trust, comfort, and willingness to adopt AVs.

A key facilitator at this level was the importance of user-centred and gender-responsive design. Women responded more positively when AV systems reflected their preferences for comfort, safety, cultural appropriateness, and personalisation. Several studies highlighted the value of customizable interfaces, well-lit interiors, and ergonomic layouts tailored to diverse user needs [38,44]. Design features such as accessible seating, non-intrusive feedback, and intuitive controls enhanced user confidence and usability, particularly for women who might otherwise feel excluded by generic or male-oriented design norms [26,44,46].

However, the lack of inclusive design also emerged as a significant barrier. Many studies reported that women felt AV interiors failed to account for their caregiving roles or expectations around hygiene, safety, and space [8,11]. Concerns ranged from uncomfortable or inaccessible seating layouts to the absence of women-only or family-friendly travel options [12,47]. The omission of gendered design considerations—such as storage space for strollers or child seats—contributed to a sense that AVs were not built with women’s needs in mind [3,13,48].

Cleanliness and environmental discomfort were also frequently mentioned. Participants expressed concerns about vehicle hygiene, odour, and maintenance, especially in shared AV contexts, citing this as a reason to avoid or distrust AV services [11,13]. The perception that AV environments might be unclean or unmanaged undermined their appeal, especially when contrasted with private vehicle use.

### Community-level influences

Community-level influences refer to the broader social, infrastructural, cultural, and environmental contexts that shape women’s access to and acceptance of AVs. These factors play a crucial role in determining whether AVs are perceived as inclusive, sustainable, and practical within everyday life.

Several studies identified sustainability and equity values as key facilitators at this level. Women respondents expressed greater openness to AVs when they were positioned as environmentally friendly or as offering enhanced access for underserved groups, including non-drivers, people with disabilities, or those living in public transit-poor areas [8,13,45,51]. In such contexts, AVs were seen not only as innovative technologies, but as tools that could align with women’s values around social responsibility and environmental impact [8].

However, these optimistic perceptions were often counterbalanced by structural and cultural barriers. The high cost of AV ownership or shared AV services was a common concern, particularly for women from lower-income households, those without secure employment, or those living in areas without competitive transportation alternatives [28,38,40,43]. In rural and peri-urban settings, participants reported limited AV infrastructure, poor road conditions, and low service availability, all of which made AVs less practical despite their potential benefits [8].

Time inefficiencies were also flagged as a deterrent. Long or rigid travel routes did not meet the complex, multitasking mobility patterns of many women, especially those engaged in caregiving, school runs, or trip-chaining errands [8,11,26]. Without flexible and responsive service configurations, AVs were perceived as insufficient for daily life.

Finally, cultural norms and social conservatism influenced perceptions in several studies. In communities with strong patriarchal values or conservative gender norms, women’s transportation choices were often shaped by concerns over visibility, propriety, and communal judgment [35,39,40,47]. Stereotypes about who “should” use new technology—combined with modesty expectations or discomfort with mixed-gender rides—undermined AV acceptance, particularly in traditional or religious contexts [13,45,51].

### Policy-level influences

Although very few studies have explored policy-related factors directly with women, we classified them as both facilitators and barriers to AV adoption. One key facilitator was the presence of supportive policies and government endorsement. When AV technology was framed within clear legal structures and national strategies, participants expressed greater trust and willingness to engage [8]. Regulatory backing was seen as a signal of legitimacy and safety, particularly for those who were otherwise uncertain about the maturity of the technology or its compliance with public standards [8].

Conversely, the unclear liability rules, limited data protection guidance, and unresolved ethical questions—such as decision-making in unavoidable crash scenarios—contributed to hesitation and scepticism about AV deployment [8]. Women expressed concerns about who would be held accountable in the event of an accident, how personal data would be handled, and whether AV systems could make ethical decisions that reflect diverse social values [8].

## Discussion

Our review places women’s experiences at the centre of analysis, addressing a notable gap in previous AV literature. While earlier review studies such as Keszey (2020) and Chen et al. (2022) offered valuable overviews of AV adoption factors, they treated gender primarily as a secondary demographic variable rather than as a central analytical lens [55,56]. For instance, although Keszey emphasised psychological and technological antecedents like hedonic motivation and technological anxiety, the framework does not explicitly address how gender roles influence these factors [55]. Similarly, Chen et al. synthesised multiple predictors of AV adoption, including demographic, policy, and psychological factors, but gave limited attention to gendered differences in perception or experience [56].

Moreover, much of the existing literature remains anchored in generalised behavioural models such as the Technology Acceptance Model (TAM) and the Unified Theory of Acceptance and Use of Technology (UTAUT). These frameworks have been instrumental in identifying core predictors like perceived usefulness and ease of use, as demonstrated in studies such as Yuen et al. (2021), which integrated TAM with Innovation Diffusion Theory to explain AV adoption [57]. However, these models often treat users as if they are all the same, overlooking how gender, caregiving roles, emotional safety, and social norms fundamentally shape technology engagement.

**Fig 8:**
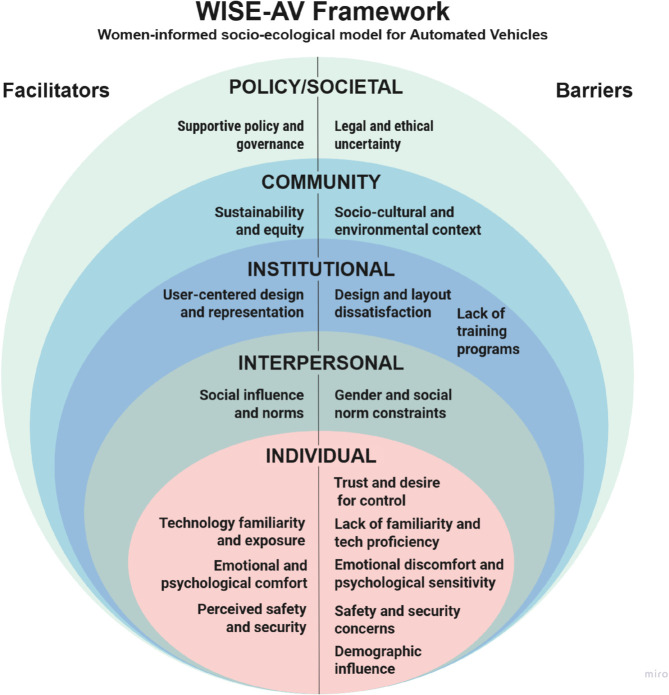
The WISE-AV framework: women-informed socio-ecological framework for automated vehicles.

This is where our contribution begins. Our review synthesises 34 peer-reviewed studies through a socio-ecological lens and offers the WISE-AV framework (Women-Informed Socio-Ecological Framework for Autonomous Vehicles) as presented in Fig 8. It recognises that women’s adoption of AVs is not simply a matter of utility or usability, but a complex negotiation of trust, safety, social expectations, and design inclusion. Our approach of mapping these influences across five socio-ecological levels — individual, interpersonal, institutional, community, and policy — WISE-AV offers a more holistic, equity-driven lens for understanding AV adoption. The following sections unpack the four core domains of this framework, illustrating how they intersect with and expand upon traditional acceptance models.

### Trust and emotional safety in AV design

For women, trust in AVs involves both operational reliability and emotional assurance. While women in the literature appreciated the rule-based logic of AVs and their potential to reduce human error and driving anxiety [26], this appreciation was often tempered by safety concerns in ambiguous or unsupervised contexts. Scenarios involving solo travel, shared rides with strangers, or lack of real-time oversight consistently triggered anxiety and hesitation [3,8,11].

Notably, these concerns persist even when AVs are promised to be technically “safe,” revealing a critical gap in how trust is conceptualised in traditional models. They expressed a strong desire for features that allow them to override decisions, track their rides, or select co-passengers—capabilities that address a need for agency, not just automation [12,30].

The absence of a human driver in shared AVs intensified feelings of vulnerability [11]. Even when safety features such as surveillance or authentication systems were present, they did not always fully offset the discomfort of being alone in a vehicle without human backup [8,13]. This underscores a fundamental flaw in AV design and communication strategies: the assumption that technical safety alone is sufficient, when in fact emotional safety is equally—if not more—important for many users. Given that the emotional dimension of AV engagement—fear, risk sensitivity, and preference for control—tends to be stronger among women than men [58], it demands a tailored communication and design approach.

The WISE-AV framework brings this nuance to the forefront. It reframes trust not as a binary variable but as a layered construct shaped by gendered experiences and emotional labour. In doing so, it challenges AV developers and policymakers to move beyond compliance checklists and toward user-informed design.

### Bridging the gap between exposure and trust

Much like trust, familiarity with technology is not a neutral or evenly distributed experience. Our review found that women’s confidence in AVs was strongly shaped by their prior exposure to related technologies—such as ADAS, simulators, or smart driving tools [41,53]. However, exposure alone was not enough. Even women who had interacted with these systems often reported low trust or limited use, particularly when the benefits were not clearly communicated or when the systems felt opaque or intimidating [37].

This is where traditional models like TAM fall short. While they acknowledge the role of perceived ease of use, they don’t account for the emotional and social scaffolding required to build that perception, especially for users who have historically been excluded from tech design and discourse. Our review, supported by Sisiopiku et al. (2023) and Havlíčková et al. (2019), shows that the type and quality of exposure matter deeply [32,41]. Women, in particular, respond more positively to immersive, participatory experiences such as driving simulators or community-based AV trials. These formats not only increase familiarity but also reduce emotional barriers and build trust. In contrast, passive exposure through media or brochures often fails to shift attitudes, especially among older women [53]. What is needed are active, participatory experiences—guided demonstrations, simulations, and community-based learning—that empower women to engage with AVs on their own terms [8,43].

The WISE-AV framework captures this nuance by situating technological familiarity within broader socio-ecological contexts. It recognises that digital literacy is not just a skill but a social condition, shaped by access, confidence, and cultural messaging. As such, it offers a roadmap for inclusive onboarding strategies that go beyond “user manuals” toward meaningful, trust-building engagement.

### Applying the WISE-AV framework to design and policy

In AV design, the challenge lies in the details, and those details too often overlook women. Across the studies we reviewed, women consistently reported discomfort with interior layouts, inadequate hygiene, and the absence of features that address caregiving roles or personal safety needs [11,13,47]. Adjustable seating, ride tracking, and female-only ride options were not “nice to haves” in shared AVs; they were deal-breakers [11,44,47].

The few studies that engaged feminist HCI approaches, such as those by Schuß et al. and Asha et al., revealed just how important it is to invite women into the design process [11,47].

The WISE-AV framework elevates this insight by embedding design equity at the institutional level. It challenges developers to move beyond “universal design” and toward responsive, context-aware systems that reflect the lived realities of diverse users.

On the other hand, broader systems such as policy, infrastructure, and cultural norms, play a decisive role in AV adoption. Our review found that women in rural areas, low-income households, or conservative communities face compounded barriers [10,43]. These include not just affordability and infrastructure, but also social scrutiny, gender norms, and a lack of policy protections [8,26]. Most AV policies remain gender-blind. Few jurisdictions have implemented or even proposed regulations that address women’s specific safety concerns or access barriers [8,51].

The WISE-AV framework addresses this directly. By mapping policy and community-level influences, it exposes how structural inequities shape individual choices. It also provides a blueprint for gender-responsive governance, encompassing training programs, legal clarity, and inclusive infrastructure planning. In doing so, it reframes AV adoption not simply as a market trend, but as a public good that must be distributed equitably.

## Limitations

Several limitations of this scoping review should be acknowledged.

First, only studies published in English were included. While this ensured consistency in interpretation and analysis, it may have excluded relevant findings from non-English-speaking regions—particularly in areas where AV research is still emerging.

Second, the database search was limited to three sources: Scopus, Web of Science, and Google Scholar. Although these platforms index a broad range of interdisciplinary and social science research, relevant studies available in other databases may have been missed.

Third, most included studies were conducted in high-income countries, which may limit the applicability of the findings to low- and middle-income contexts.

Fourth, the final sample size of 34 studies reflects the limited availability of research that centres on women in AV contexts. This reinforces the need for further primary research that applies a gender-focused lens.

Fifth, the review did not include technical or policy-modelling studies that lack gender-differentiated outcomes, even though such work may indirectly influence women’s AV experiences (e.g., through infrastructure, insurance, or deployment models). Future reviews could explore these macro-level factors in relation to equity and AV adoption.

Finally, despite efforts to capture a broad range of perspectives, there remains a lack of representation of older women, women from marginalised communities, and those with intersecting identities in the included literature. Addressing these gaps presents an opportunity for future research to strengthen the inclusivity and equity dimensions of AV adoption studies.

## Conclusion

This scoping review synthesised 34 studies (2016–2025) on women’s experiences with automated vehicles (AVs). While gender is often recorded as a demographic in AV research, women are rarely centred as primary users or invited to co-design vehicles, interfaces, services, or deployment plans. As a result, women’s day-to-day needs, such as safety, caregiving logistics, comfort, and route flexibility, are underrepresented in what is ultimately built. This oversight isn’t just a missed opportunity for equity but also for improving the effectiveness and adoption of AV technologies in real-world settings.

Most of the reviewed studies relied on quantitative methods and mixed-gender samples. However, many factors shaping women’s mobility—such as caregiving roles, emotional safety, and cultural expectations—require deeper qualitative and participatory engagement, as they often remain invisible in traditional, male-dominated transport research. Without inclusive data and representative voices, current AV systems risk reinforcing, rather than resolving, existing transport inequities.

To address this, we propose the WISE-AV Framework: a Women-Informed Socio-Ecological Model that maps real-world barriers and enablers of AV adoption across five levels—individual, interpersonal, institutional, community, and policy. This is more than a framework; it is a call to action. WISE-AV helps researchers, designers, and policymakers understand how emotional risk, design exclusion, social norms, and policy gaps intersect to shape women’s mobility choices.

Theoretically, this work extends the Socio-Ecological Model into the AV domain through a gender lens, drawing on feminist HCI principles such as transparency, participation, and embodied experience. Practically, it urges the co-creation of AV systems that reflect women’s trust needs, caregiving responsibilities, and mobility patterns—not as an afterthought, but as a foundation. Methodologically, it demonstrates how combining a scoping review with reflexive thematic analysis can surface not only trends but also silences—those critical gaps where women’s voices should be.

Ultimately, AVs will only be truly transformative if they are designed not for a generic “user,” but with the diverse realities of women in mind. The WISE-AV framework offers a roadmap to that future—one where technology advances not only mobility but also equity.

## Supporting information

S1 File This file contains the study characteristics of the reviewed studies.

(XLSX)
